# Alkali Concentration and Diluent Effects on Properties of Grape Cane Fiber-Reinforced Polymer Composites

**DOI:** 10.3390/polym13234055

**Published:** 2021-11-23

**Authors:** Balkis F. A. Bakar, Frederick A. Kamke

**Affiliations:** 1 Department of Wood and Fiber Industry, Faculty of Forestry and Environment, Universiti Putra Malaysia, Serdang 43400, Selangor, Malaysia; 2 Wood Science and Engineering, College of Forestry, Oregon State University, Corvallis, OR 97333, USA; fred.kamke@oregonstate.edu

**Keywords:** unsaturated polyester, acrylated epoxidized soybean oil, styrene, alkali treatment, grape cane fibers, agrofibers

## Abstract

The main objective of this study was to investigate the properties of polymer composites reinforced with grape cane fibers. The fibers were subjected to a sodium hydroxide (NaOH) treatment at two treatment concentrations to extract the fibers as well as fiber surface treatment. Panels were fabricated by hand lay-up and compression molding according to different fiber types, namely outer bark (OB) and whole (W) fibers. The whole fiber was a mixture of OB and inner bark (IB) fibers. Grape cane fibers were used as the reinforcement material for unsaturated polyester (UPE) resin panels. Acrylated epoxidized soybean oil (AESO) was used as a reactive diluent material with the UPE resin, and the results were compared with panels prepared with commercial styrene–UPE. There were inconsistent alkali treatment concentration effects on the mechanical properties and water absorption. However, panels fabricated with the whole bark fibers that have been treated with 1 wt % NaOH and had AESO–UPE resin resulted in the best tensile and flexural strength.

## 1. Introduction

The effort to use bio-based material in composite applications was awakened in the 1970s after the oil crisis and continues until today. In the case of fiber-reinforced polymer composites, industries and academia have done numerous studies to replace one or more components with bio-based materials. A biocomposite is a term used to describe a composite material made up of one or more phases of bio-based materials that can be used in the reinforcement or matrix phases. For instance, a composite that is fabricated with a plant fiber as the reinforcement material in a polymer matrix is called biocomposite or plant fiber-reinforced polymer (PFRP). Similarly, a matrix that is synthesized from at least one natural-based material can be categorized as a biocomposite or biopolymer [1].

PFRP is commonly used for commercial non-structural applications in the automotive industry for parts, such as door panels, seat backs, dashboards, and many more [2]. Demand PFRP has been increasing each year since 2000. The demand has been driven mainly by increasing public awareness of sustainability as well as interest in safer fabrication processes. The polymer composite industries have been dominated by glass fiber-reinforced polymer (GFRP) composites. The GFRP composites, with unsaturated polyester (UPE) resin, are used in automobile, marine, wind power, and other industries [3,4] because they offer high mechanical properties and durability [5,6]. However, GFRP composites have several drawbacks, such as high density, poor recycling, and are non-biodegradable. Therefore, research on PFRP alternatives, which offer low density and biodegradability due to its plant fiber components [7,8,9], has been aggressively explored.

Demand for PFRP composites is increasing due to the advantages of renewability, low density, availability, lower environmental impact, lower cost, and high specific strength and modulus [10,11,12]. Plant fiber also inherits hygroscopicity behavior due to the hydroxyl groups in the cell wall that influence the interfacial adhesion between fiber and resin, which is a major obstacle. However, fiber surface modification, such as alkaline treatment, can improve compatibility and improve the overall properties of PFRP [10,13,14,15].

The UPE–thermoset resin is the most common polymer used for GFRP and PFRP—accounting for over 80% of the market [16]. Industrial UPE resins are typically synthesized from diols (propylene glycol (PG)), unsaturated dibasic acids or acid anhydrides (isophthalic acid (IPA)), and saturated dibasic acids or acid anhydrides (maleic anhydride (MA)) [17,18,19,20]. A crosslinking agent or diluent needs to be introduced to the UPE to react with the C=C bonds and increase chain mobility of UPE chains, thus reducing the tendency of homopolymerization [20].

The most common diluent is styrene, which constitutes about 35–50 wt % of UPE resins [19]. Styrene has a high vapor pressure at room temperature. It is reported to cause multiple health hazards, such as skin and respiratory irritation [19].

One alternative to styrene is acrylated epoxidized soybean oil (AESO), which is commercially available and has successfully replaced styrene-based UPE resin for both GFRP and PFRP composites [17,18,20,21]. A comparison of the mechanical properties between AESO–UPE–kenaf composite and styrene–UPE–kenaf composite showed that the results were similar or even superior for the AESO–UPE–kenaf composite [20].

A potential plant fiber for PFRP is grape cane, which is a by-product of a vineyard that is available after annual pruning. There are also other by-products generated from the vineyard, especially after winemaking processes such as grape pomace that consists of seeds, skins, and the remaining pulps [22]. This type of by-products can be converted to livestock feed, flour, and many others [23]. However, in the current study, the grape cane is used for the potential raw material for PRFP. Approximately 90% of the canes are removed [24] to generate approximately five oven-dry tons per hectare [25,26]. Pruning residues are currently either mulched or piled and burned, with costs ranging from 50 to 200 Euros per hectare [27]. Therefore, the main objective of the current study was to evaluate the technical feasibility of PFRP fabrication made of grape cane fiber and UPE, focusing mainly on the tensile, flexural, and water absorption properties.

## 2. Materials and Methods

### 2.1. Grape Cane Fibers

**Figure 1:**
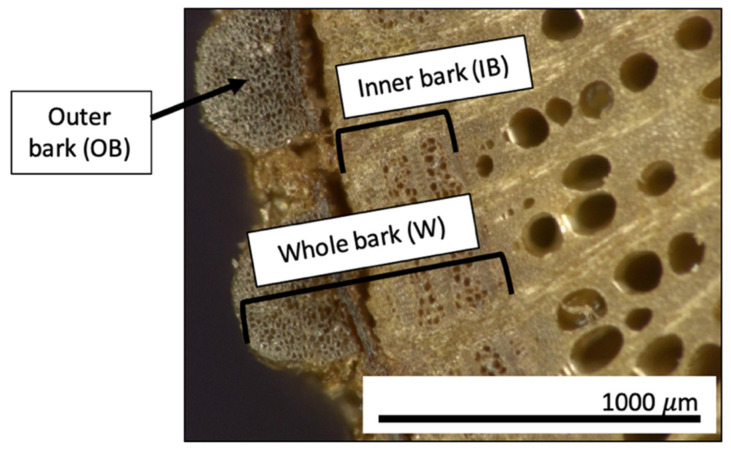
Cross-section of grape cane captured using a digital microscope, Keyence (Itasca, IL, USA).

Figure 1 shows the cross-section of a grape cane stem. Two types of bark fibers were used to fabricate the grape cane composite panels, namely outer bark (OB) fibers and a mixture of OB and inner bark (IB) fibers, which were labeled as OB and whole (W) fibers, respectively. The OB fibers were stripped manually from the cane and treated separately. To obtain the whole fibers, the whole cane was treated to separate the whole bark fibers from the wood part. An industrial operation would likely use whole fibers because the process would be easier, and the fiber yield would be greater.

### 2.2. Fiber Treatment

Alkali treatment was carried out to separate these fibers into smaller fiber bundles. Two different alkali concentrations were used in the current study: 1 and 3 wt % NaOH (Sigma-Aldrich, St. Louis, MO, USA) and were labeled as N1 and N3, respectively. The fibers were placed in a flask filled with alkali solution at 100 °C for 2 h. Then, the fibers were rinsed with running hot water until pH 7 was obtained, as determined using pH strips. After rinsing, the fibers were left to air-dry for 24 h and then dried in an oven at 60 °C until a constant weight was obtained. The target moisture content was below 10%.

### 2.3. Fiber Mat Preparation

**Figure 2:**
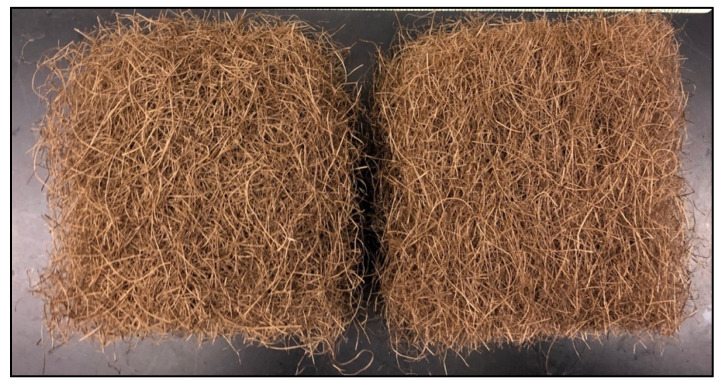
Example of fiber mats prepared from (**left**) OB fibers and (**right**) whole fibers of grape cane fibers.

Fiber mats from each group of fibers were prepared separately using a Louet drum carder (Louët, Lochem, Netherlands). The OB fibers were fed into the drum carder to separate larger fiber bundles from smaller bundles through carding, layering, and needle-punching processes. A long fiber mat was formed and was further cut into four small square mats with a size of 100 mm × 100 mm by a paper cutter (X-ACTO Model 1515G, Westerville, OH, USA). Then, these mats were oven-dried at 103 °C for at least 24 h prior to fabrication. The total weight of each fiber mat was 8 ± 0.05 g. The same process was repeated for whole fibers, as shown in Figure 2.

### 2.4. Resin

Acrylated epoxidized soybean oil (AESO) containing 4000 ppm monomethyl ether hydroquinone and *tert*-butyl peroxybenzoate (TBPB) were purchased from Sigma-Aldrich (St. Louis, MO, USA). The UPE used in this study was propylene glycol–isophthalic acid–maleic anhydride (PG-IPA-MA) plastic and the styrene–(PG-IPA-MA) resin, which were obtained from Ashland Inc. (Dublin, OH, USA).

### 2.5. Resin Preparation

The preparation of resin was adapted from the method of Wu and Li (2016). AESO, with a weight of 12 ± 0.01 g, was placed in a 150 mL beaker and heated to 90 °C in an oil bath. Then, 8 ± 0.01 g of ground UPE was slowly added into AESO and stirred for 10 min to generate a homogenous and pourable resin. Then, the resin mixture was purged with nitrogen for three min and cooled to 70 °C. Afterward, TBPB was added based on 1.5 wt % of the total weight of the resin and stirred for an additional 3 min. The resulting AESO–UPE resin had a ratio of 60:40 and was labeled as AESO–UPE. The commercial styrene–UPE was mixed with 1.5 wt % TBPB and labeled as styrene–UPE. Wu and Li (2016) determined the resin viscosity of the same resin formulation used in this study to be 10 Pa s at 56 °C, 3 Pa s at 70 °C, and 1 Pa s at 80 °C. At 105 °C, the viscosity began to increase rapidly, and based on their Differential Scanning Calorimetry (DSC) results, the resin gels at 110 °C.

### 2.6. Fabrication of Grape Cane Fiber-Reinforced Composites

The ratio of the fiber to the resin used in the current study was 40:60. The AESO–UPE resin was kept in a 70 °C water bath during the fabrication process to maintain low viscosity, while the styrene–UPE resin was stored at 40 °C. Teflon film, with 0.1 mm thickness, was placed in a steel mold prior to adding the 8 g oven-dry fiber mat. The mold dimension was 100 mm × 100 mm × 2 mm (length × width × depth). A total of 12 g of resin was poured onto the fiber mat, and then, a stainless-steel compression plate was placed on top. The complete mold, with resin-saturated fiber mats, was pressed at 3.5 MPa for 10 min at 20 °C with Versa Tester press (Fred S. Carver Inc., Menomonie, WI, USA). Then, the mold was transferred to an automatic Carver press (Carver Inc., Wabash, IN, USA) at a temperature of 160 °C and pressed at 4.5 MPa for 45 min. Afterward, the mold was transferred to the cold press under the same pressure to allow a slow cooling process for 100 min. The resulting panels were stored at room temperature for 24 h before testing.

Control samples containing neat resin without fiber reinforcement were fabricated. The panels were labeled according to the combination fiber and resin types. For instance, control samples for AESO–UPE resin were labeled as CA, and control samples for styrene–UPE resin were labeled CS (Table 1). SO-N1 panels were fabricated with styrene-based resin with OB fibers that were treated with N1 treatment (1 wt% NaOH).

### 2.7. Mechanical Testing

Tensile tests were carried out following ASTM D3039 (2017). A dumbbell shape, with dimensions 58 mm by 14.5 mm by 2 mm, was cut from the panel. The gage length of the test specimen was 30 mm, and the grip lengths were 11 mm on each end. The specimen was tested at a crosshead speed of 0.5 mm/min. A digital image correlation (DIC) system was integrated with the tensile test to analyze the elongation during the test. The tensile test specimen was coated with black paint, and then, a white speckle pattern was deposited onto the specimen using an air gun to create a random speckle pattern. The captured DIC images during the test were analyzed using VID-2D analytical software (Correlated Solutions, Columbia, SC, USA) as reported by others [28,29]. There were six replications per treatment.

A test specimen with a dimension of 65 mm by 12.7 mm by 2 mm was prepared for the flexural test according to ASTM D790 (2017). The specimen was placed horizontally on two supports at a span length of 50 mm and a crosshead speed of 0.6 mm/min. There were six replications per treatment.

### 2.8. Water Absorption

The dimensions of the water absorption test specimens were 38 mm by 12.7 mm by 2 mm, and the test was conducted according to ASTM D570 (2018). The specimens were initially dried in an oven for 24 h at 50 °C, cooled in a desiccator, and weighed before the test. Then, the specimens were immersed in distilled water at room temperature. The specimens were removed from the water at the designated time, wiped with dry tissue papers, weighed, and replaced in the water until the weight difference within a two-week period was less than 1%. There were six replications per treatment.

### 2.9. Scanning Electron Microscopy

The effect of diluent types on the morphologies of grape cane fiber-reinforced composites was observed. Specimens from the tensile tests were examined according to the panel types using an FEI QUANTA (Thermo Fisher Scientific, Hillsboro, OR, USA) environmental scanning electron microscopy (SEM). The specimens were carbon taped to aluminum stubs with care taken to ensure that the fracture surface was facing upwards. Then, the mounted samples were sputter-coated with gold/palladium for 35 s. A high vacuum and voltage of 5 kV were applied during image collection.

### 2.10. Statistical Analysis

The data were statistically analyzed using R studio software (RStudio, Boston, MA, USA), Version 1.3.1093. Analysis of variance (ANOVA) was used to examine the effects of treatment concentrations and resin types on the mechanical properties and water absorption of grape cane fiber-reinforced polymer composites. Tukey’s honest significant difference (Tukey HSD) test was used for further evaluation of the effect of the main parameter on each test. Tukey HSD ranks the means and calculates the minimum value to be significantly different from each other at *p* < 0.05. Means followed by the same letter (a, b, c, etc.) are not significantly different. The effect of fiber treatment on mechanical properties was analyzed separately for each panel type. In addition, fiber treatments were pooled to compare across panel types.

## 3. Results and Discussion

### 3.1. Mechanical Properties

The overall effect of treatment concentrations on the tensile and flexural properties of grape cane was evaluated according to the results presented in Table 2. When comparing the control samples, which were pure polymer panels, panels with styrene–UPE (CS) had higher mechanical properties than panels fabricated from AESO–UPE (CA). Panels reinforced with grape cane fibers showed comparable tensile properties to CS panels and consistently higher tensile properties than CA panels, regardless of the resin and fiber types. There was a slightly different trend observed in flexural properties. Grape cane fiber panels had slightly lower flexural properties than CS panels, but higher than CA panels. The results showed that grape cane fibers can be an effective reinforcement material for the polymer systems in this study because they improved some properties of the panels and reduced the amount of resin used.

The influence of fiber treatment concentrations is not apparent for most of the measurements except for the tensile strength of AW and SO panels. Alkaline treatment was performed to separate the fibers from the core part of the grape cane and also as a fiber modification to reduce the hydrophilicity of the plant fibers. This treatment has been extensively studied and exhibited positive results to increase the interfacial adhesion between plant fiber and resin in a composite panel [14,30,31]. Based on results in Table 2, treatment with 1% NaOH was sufficient to improve performance, and no further benefit was realized at 3% NaOH.

### 3.2. Influence of Resin Types on Mechanical Properties of Grape Cane Fiber Panels

#### 3.2.1. Resin Control Panels

**Figure 3:**
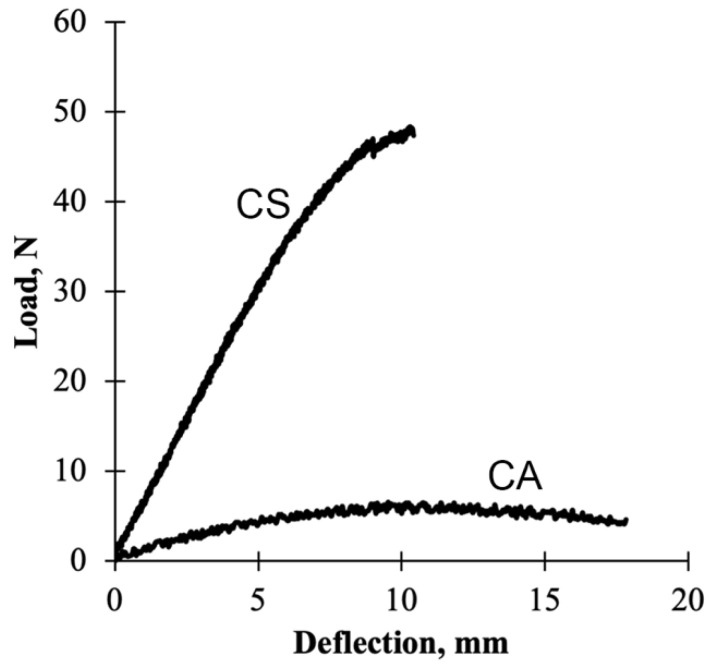
An illustration of load–deflection curve for flexural test of CA and CS panels.

**Figure 4:**
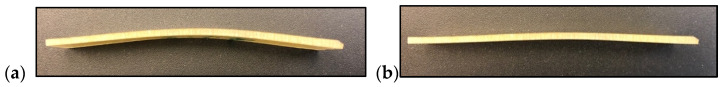
Tested flexural specimen of CA panels, (**a**) 0 min after the test, and (**b**) 5 min after the test.

Figure 3 presents a load–deflection curve of the flexural test for both control resin panels. Noticeably, the behavior of these resins is different. For instance, at the same test condition, the CA panels are more ductile and did not have abrupt failure, whereas the CS panels were more brittle and exhibited abrupt failure. The CA panels continued flexing during the test until the test was manually stopped. After the load was removed, with some time, the shape of the CA panels returned to their nearly original shape (Figure 4).

The flexural behavior of UPE resin is heavily reliant on the styrene content. Sanchez et al. (2000) reported that a 6% styrene content in UPE resin resulted in more ductile behavior when compared to UPE resin with 38% styrene content. Similar flexural behavior was observed from the current study where the CS panels have significantly higher modulus compared to the CA panels, which had no styrene content.

**Figure 5:**
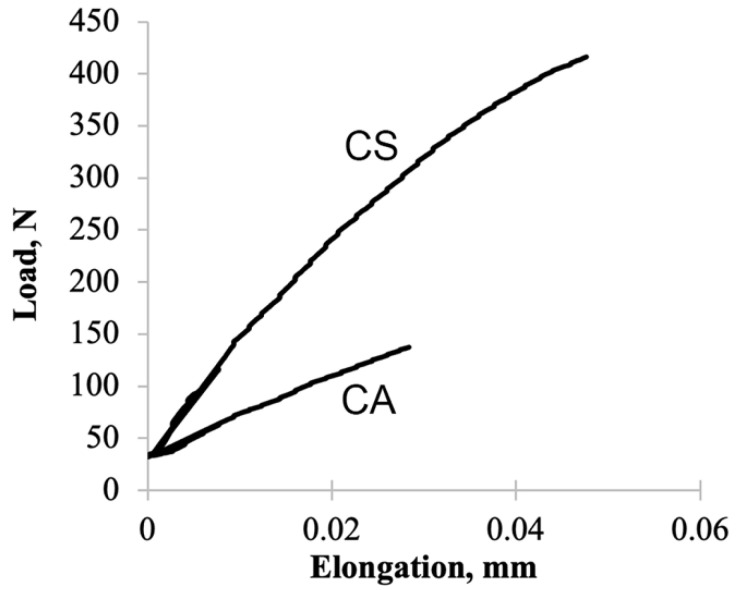
An illustration of load–deflection curve for tensile test of CA and CS samples.

Similar to flexural behavior, the CS panels have better tensile properties than CA panels, with CS panels having four times higher tensile load at failure than CA panels (Figure 5). Both resins have small elongation showing their brittle properties; however, CA panels have lower modulus and shorter elongation if compared to CS panels.

The lower mechanical properties of CA panels may be due to the higher molecular weight of AESO (≈1260 g/mol), which is approximately 12 times higher than styrene (104 g/mol). C=C bonds in AESO have large steric hindrance, which limits mobility as well as limits the potential for crosslinking with UPE [20]. In contrast, the C=C bonds in styrene are more accessible for crosslinking, thus promoting a more rigid polymer structure. This limitation may result in lower crosslink density between AESO and UPE than styrene and UPE, thus contributing to the lower mechanical properties of the CA panels. Entanglements between polymer chains will increase the modulus, and greater molecular weight increases the probability of entanglement, thus suggesting the potential for AESO to form a more rigid polymer than styrene. The distance between entanglements or crosslinks will greatly affect the modulus, and this information is lacking in the current study. However, crosslinking is more effective than entanglements to enhance modulus given otherwise consistent polymer morphology [32,33]. The styrene–UPE system is far more likely to crosslink than the AESO–UPE system [20].

#### 3.2.2. Grape Cane Fiber-Reinforced UPE Resin Composites

Regardless of the resin type, there was no statistical difference between the panels in all cases of grape cane fiber-reinforced UPE resin composites, with the exception of AO tensile modulus. In general, among the three panel types, when the control samples were excluded, AW panels exhibited the highest mean in tensile and flexural strength, while SO panels displayed the highest mean in tensile and flexural moduli (Table 3).

Assuming adequate adhesion, adding reinforcement material to a resin matrix typically improves the overall performance of the composite. In some cases, fiber addition increases the modulus but reduces the strength [34]. The mechanical properties of AESO-based grape cane panels were improved compared to CA specimens, as displayed in Table 4. For instance, the greatest improvement was observed in both tensile and flexural moduli for AO and AW panels (Table 4). For instance, AO-N1 panels had a 625% and 633% increase for the tensile and flexural moduli, respectively, compared to control specimens; while AW-N1 panels had approximately 775% and 667% increase of tensile and flexural moduli, respectively, when compared to CA panels.

- AESO–grape cane panels

Comparing AO and AW panels without control samples shows that the AW panels had a slightly higher mean for both tensile and flexural properties (Table 3); however, there was no statistical difference except for tensile modulus. No treatment concentration effects on the properties of these two panels were observed.

**Figure 6:**
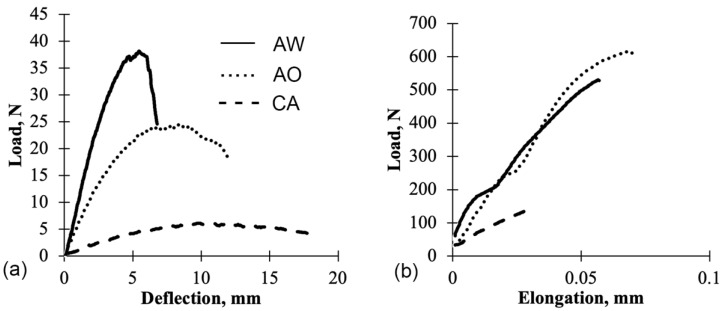
Comparison of load–deflection curves of AO, AW, and CA panels: (**a**) Flexural test and (**b**) Tensile test.

Figure 6 shows an example of load–deflection curves of the flexural and tensile tests for AESO-based panels with and without fiber reinforcement (CA, AW, AO). Based on this example, adding the whole bark fibers drastically improved the flexural properties. Whole bark fibers and outer bark fibers had a similar effect on the tensile properties.

The AW panels were fabricated from a mixture of the outer and inner bark of grape cane (Figure 1). Although these fibers originated from the same area (bark area), they have distinctive morphological cell units and different chemistry [29,35,36,37]. Due to these differences, one would expect that the fibers may have different behavior under compression during composite fabrication. For instance, the combination of these fibers in the composite panels appears to complement each other in terms of having better packing. Better fiber packing in the AW panels may be due to the difference in the cross-section and the texture of these fibers [37], resulting in better overall properties, depending on the NaOH treatment.

- Styrene-Based Panels

Adding grape cane fibers in styrene-based resin panels resulted in less or equal values for tensile and flexural properties, respectively (Table 5). Styrene–UPE resin is a commercial product with a wide range of applications, including automotive, marine, and construction. It offers high mechanical properties and durability. The reduction in flexural properties upon the addition of grape cane fiber may be due to the weak interface adhesion between fiber and the resin. No treatment concentration effects were observed in SO panels, except for flexural strength (Table 2). The finding showed that N1 may be sufficient to fabricate panels with better overall performances.

**Figure 7:**
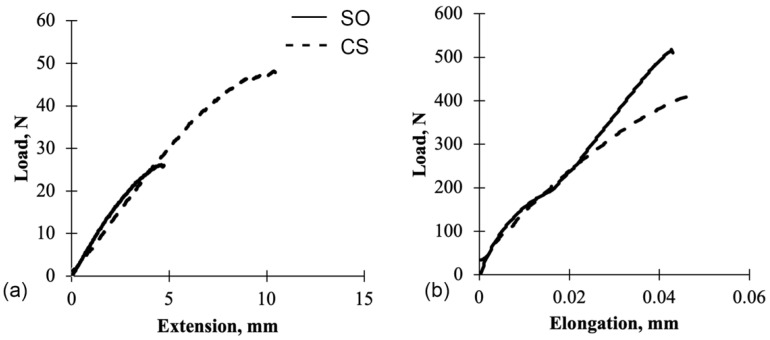
Comparison of load–deflection curves of SO and CS panels: (**a**) Flexural test and (**b**) Tensile test.

Figure 7a,b show examples of load–deformation curves for flexural and tensile tests of styrene-based panels (CS and SO). CS panels had approximately two times more deflection for the flexural test and had approximately the same elongation as the SO panels for the tensile test. According to Table 2, when grape cane fibers were added to the styrene-based panels, the mechanical properties were comparable to the control panels. The findings show that using grape cane fibers in the composite fabrication reduces the resin content, but there is no improvement of properties.

In general, the grape cane composites had relatively similar mechanical performance to other PFRP products reported in the literature but lower performance than comparable GFRP products [38,39,40]. Past studies have explored underutilized agricultural wastes, such as rice husks, bagasse, vakka, banana, and pineapple leaves [40,41,42,43] and other non-wood fibers, such as kenaf, bamboo, Napier grass fiber, and jute [20,39,41,44] in composite applications. Composites made with commercial non-wood fibers have higher overall composite properties when compared with fibers from underutilized agricultural wastes [20,41,45]. For instance, untreated kenaf fiber-reinforced styrene–UPE resin panels had values of approximately 105 MPa and 4.5 GPa for tensile strength and tensile modulus, respectively [20]. In comparison, alfa fiber composite panels had a tensile strength and tensile modulus of 17.5 MPa and 0.4 GPa, respectively [13]. Another study reinforced the polyester resin with Napier grass fibers and reported the tensile strength and tensile modulus of 13 MPa and 1.5 GPa, respectively [44]. For panels with glass fibers, the tensile strength and tensile modulus were 110 MPa and 6.5 GPa, respectively [38]. Although the findings from the current study did not exceed the performance of GFRP or PFRP made from commercial non-wood fibers, when compared to PFRP from underutilized fibers, the grape cane fibers exhibited better properties.

### 3.3. Water Absorption

**Figure 8:**
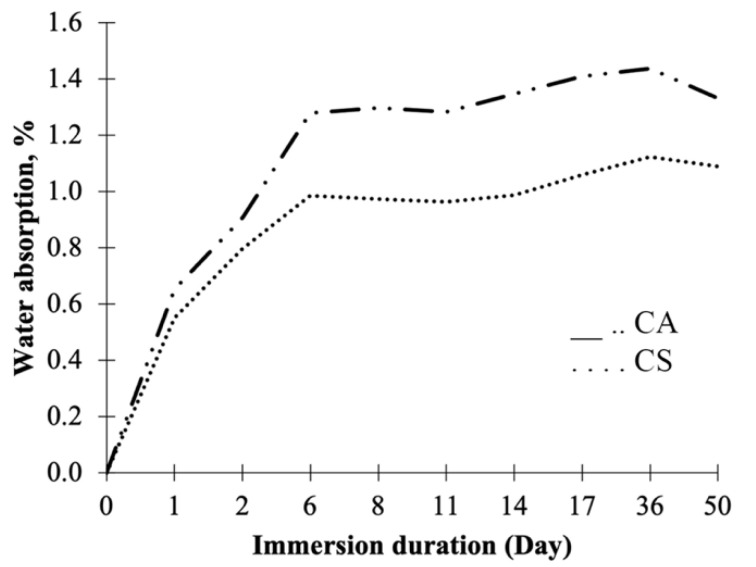
Water absorption of AESO–UPE (CA) and styrene–UPE (CS) panels.

The water absorption (WA) of CA and CS panels is presented in Figure 8. Regardless of the resin type, the water uptake of both control panels increased with immersion time. Control panels with AESO–UPE resin (CA) had a statistically higher water uptake by 22% than styrene–UPE (CS) panels at the end of the test cycle. The greater WA of the CA panels may be due to the higher molecular weight AESO and greater steric hindrance from its C=C bonds compared to styrene. The resulting AESO–UPE polymer would have less crosslinking and less packing [21], thus allowing more space for water absorption compared to styrene–UPE.

A similar water absorption trend was observed when grape cane fibers were added. All panels had a rapid increase in water absorption up to Day 6; then, there was a gradual increase until leveling out when approaching the end of the test cycle. In general, panels fabricated with grape cane fibers and styrene–UPE had slightly lower WA when compared to fibers–AESO–UPE panels (Table 6). However, no significant differences were detected between the three panel types at the end of the immersion period. In addition, from observation, some material leached out from all test specimens during the test that might create an opening for water absorption.

Panels treated with N3 had significantly higher water uptake (17.4%) when compared to N1-treated panels at the end of the test cycle when comparing the treatment concentration effects on the AESO panels. Theoretically, a reduction in the hydrophilicity properties of the fiber can be expected due to alkaline treatment at an optimum concentration [15,31]. However, N3 treatment seemed to have an adverse effect on the WA of AESO panels. This may be caused by an excessive removal of lignin during the treatment, together with the hemicelluloses, interfering with bonding sites with the resin. In addition, the cellulose regions may have become more accessible during the WA tests. Conversely, there was no significant difference in water uptake between treatment concentrations observed for SO panels. It is typical to have higher water uptake in panels reinforced with plant fibers in comparison to pure resin panels [7,12,39,46]. It is well-known that plant fibers are hydrophilic. Additionally, as the fiber content increases, the water uptake increases. About 33% water gained when adding an additional 15% of flax fiber in fiber tensile specimens was reported by [47]. Additionally, the water uptake may enter the panels through gaps at the fiber–resin interface, cracks in the resin, as well as voids in the panels.

### 3.4. SEM Images

**Figure 9:**
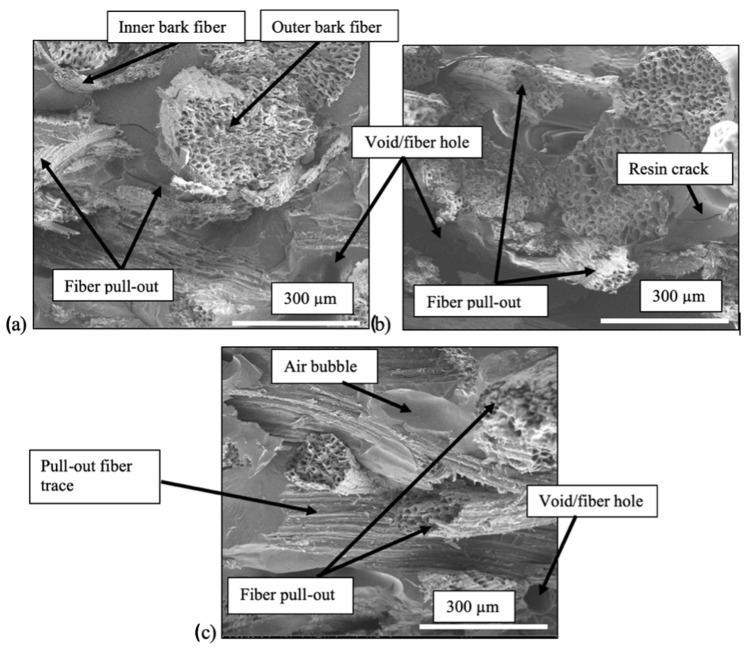
SEM images of tensile fractured sample of (**a**) AW panel, (**b**) SO panel, and (**c**) AO panel.

After the tensile test, the fractured surfaces were examined under SEM to study the fiber–matrix adhesion for all panels. In general, there is no strong distinction in interfacial adhesion among the panels and the treatment concentrations. Some fibers failed near the fracture surfaces observed for all panels, which demonstrated good adhesion between fiber and resin. However, there was also evidence of fiber pull-out, which suggests weak interfacial interaction between fiber and resin. This observation occurred regardless of the panel type (Figure 9). In addition, cracks in the resin and voids were present occasionally in all panels. Comparable observations were reported in several studies using different types of fibers and resin combinations [48,49,50,51].

**Figure 10:**
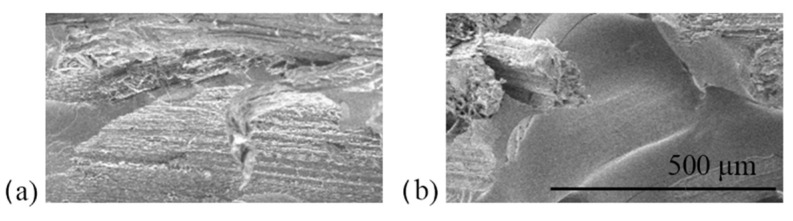
Fiber pull-out traces in (**a**) Rougher fiber trace on AESO–UPE panels and (**b**) Smooth fiber trace on styrene–UPE panels.

**Figure 11:**
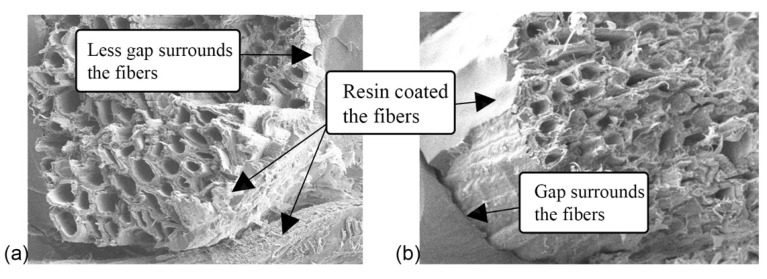
SEM images of fracture surface and fiber breakage; (**a**) AESO–UPE and (**b**) Styrene–UPE.

Another noteworthy observation under SEM is the surface of the pull-out fiber traces. The fiber pull-out surfaces were rougher for AESO–UPE panels compared to styrene–UPE panels, as shown in Figure 10. Styrene–UPE panels have more clean fiber hole traces (Figure 11b) than AESO–UPE panels (Figure 11a). This may show that the AESO–UPE resin had better fiber wetting than the styrene–UPE resin, which resulted in a better performance of composite panels in general.

Figure 11 shows the interface between N3-treated fibers in AESO–UPE (AW panels) and styrene–UPE panels. It was observed that more gaps were surrounding the fibers in styrene–UPE panels than in the AESO–UPE panels. There was also evidence that both resins coated the fibers in the SEM images, which supports the mechanical results in the current study.

## 4. Conclusions

The current study successfully fabricated grape cane fiber-reinforced unsaturated polyester composites. The technique to separate the fibers from the cane was carefully considered. In this regard, fibers were treated at different concentrations of sodium hydroxide. The effect of treatment concentration and origin of the fibers within the cane (outer bark and inner bark) were investigated. In addition, in an effort to reduce harmful styrene as the reactive diluent, a bio-based replacement material was also studied. Tensile and flexural properties, as well as water absorption of the panels were measured and compared with the control panels for each resin formulation.

The control resin panels made of AESO–UPE had lower mechanical properties and higher water absorption when compared to the control resin panels made of styrene–UPE. As expected, the water absorption of the panels increased when plant fibers were incorporated. No differences in mechanical properties and water absorption were observed between AO, SO, or AW panels, which indicates that whole fiber and outer bark fiber had similar effects. In general, grape cane fiber increased the flexural and tensile properties when added to AESO–UPE but had no effect on tensile properties, and it had a negative effect on flexural properties when added to styrene–UPE. Treatment effects were more noticeable on the AO and AW panels than SO panels, particularly for tensile strength and water absorption.

## Figures and Tables

**Table 1:** Designated labels for panels.

Fiber	Resin	Label
Outer bark	Styrene–UPE	SO
Outer bark	AESO–UPE	AO
Whole bark	AESO–UPE	AW
-	AESO–UPE	CA
-	Styrene–UPE	CS

**Table 2:** Tensile and flexural properties of grape cane fiber composite panels according to the resin type and fiber treatment.

Panel	Treatment	Tensile		Flexural	
		Strength, MPa	Modulus, GPa	Strength, MPa	Modulus, GPa
AO	CS	23.7 a	3.2 a	90.7 a	3.2 a
		(0.8)	(1.0)	(9.9)	(0.2)
	CA	9.1 b	0.4 b	12.6 c	0.3 c
		(2.3)	(0.2)	(5.5)	(0.1)
	N1	23.8 a	2.9 a	45.3 b	2.2 b
		(3.3)	(0.9)	(7.1)	(0.6)
	N3	22.1 a	2.7 a	43.0 b	2.4 b
		(4.8)	(0.9)	(8.6)	(0.7)
AW	CS	23.7 b′	3.2 a′	90.7 a′	3.2 a′
		(0.8)	(1.0)	(9.9)	(0.2)
	CA	9.1 c′	0.4 b′	12.6 c′	0.3 c′
		(2.3)	(0.2)	(5.5)	(0.1)
	N1	22.3 b′	3.5 a′	50.5 b′	2.3 b′
		(9.3)	(0.5)	(6.8)	(0.4)
	N3	33.8 a′	3.3 a′	50.0 b′	2.3 b′
		(6.2)	(0.3)	(12.5)	(0.6)
SO	CS	23.7 a″	3.2 a″	90.7 a″	3.2 a″
		(0.8)	(1.0)	(9.9)	(0.2)
	CA	9.1 c″	0.4 b″	12.6 c″	0.3 c″
		(2.3)	(0.2)	(5.5)	(0.1)
	N1	22.3 b″	3.8 a″	44.3 b″	2.5 b″
		(3.8)	(0.7)	(5.8)	(0.4)
	N3	33.8 a″	3.7 a″	48.4 b″	3.0 b″
		(6.3)	(0.4)	(9.5)	(0.7)

Data are expressed as mean and SD (in the parentheses) based on six measurements. Means followed by the same letter are not significantly different at *p* ≤ 0.05. An additional symbol after the letters indicates that separate statistical analysis was performed for each panel type.

**Table 3:** Mean tensile and flexural properties of grape cane fiber composite panels according to the resin type, excluding the control samples.

Panel	Tensile		Flexural	
	Strength, MPa	Modulus, GPa	Strength, MPa	Modulus, GPa
AO	22.9 a	2.8 b	44.1 a	2.3 a
	(4.0)	(0.8)	(7.6)	(0.6)
AW	28.0 a	3.4 a	50.2 a	2.3 a
	(9.7)	(0.4)	(9.6)	(0.5)
SO	25.0 a	3.7 a	46.4 a	2.8 a
	(5.0)	(0.6)	(7.8)	(0.6)

Data are expressed as mean and SD (in the parentheses) based on six measurements. Means followed by the same letter are not significantly different at *p* ≤ 0.05.

**Table 4:** Percent increase of tensile and flexural properties of AO and AW panels when compared to CA panels.

Panel	Treatment	Tensile		Flexural	
		Strength	Modulus	Strength	Modulus
AO	N1	162	625	260	633
	N3	143	575	241	700
AW	N1	145	775	301	667
	N3	271	725	297	667

**Table 5:** Percent increase of tensile and flexural properties of SO panels when compared to CS panels.

Panel	Treatment	Tensile		Flexural	
		Strength	Modulus	Strength	Modulus
SO	N1	(6)	19	(51)	(22)
	N3	43	16	(47)	(6)

Value in parentheses means CS panels have higher properties.

**Table 6:** Percentage of water absorption of AO, AW, and SO panels at the end of the immersion period (Day 50).

Panel/Treatment	AO	AW	SO
CA	1.3 c	1.3 c	1.3 b
	(0.2)	(0.2)	(0.2)
CS	1.1 c	1.1 c	1.1 b
	(0.1)	(0.1)	(0.1)
N1	33.7 b	38.0 b	36.4 a
	(4.0)	(1.1)	(2.9)
N3	41.9 a	42.3 a	35.8 a
	(1.5)	(3.1)	(5.1)

Data are expressed as mean and SD (in the parentheses) based on six measurements. Means followed by the same letter are not significantly different at *p* ≤ 0.05.

## Data Availability

The data that support the findings of this study are available from the corresponding author, upon reasonable request.
